# Multi-pulse laser-induced bubble formation and nanoparticle aggregation using MoS_2_ nanoparticles

**DOI:** 10.1038/s41598-020-72689-x

**Published:** 2020-09-25

**Authors:** Brian Ko, Weigang Lu, Alexei V. Sokolov, Ho Wai Howard Lee, Marlan O. Scully, Zhenrong Zhang

**Affiliations:** 1 Baylor University, Waco, TX 76798 USA; 2 Texas A&M University, College Station, TX 77843 USA; 3 University of Irvine, Irvine, CA 92697 USA; 4 Princeton University, Princeton, NJ 08544 USA

**Keywords:** Condensed-matter physics, Materials for optics, Nanoparticles, Optical manipulation and tweezers

## Abstract

Understanding of how particles and light interact in a liquid environment is vital for optical and biological applications. MoS_2_ has been shown to enhance nonlinear optical phenomena due to the presence of a direct excitonic resonance. Its use in biological applications is predicated on knowledge of how MoS_2_ interacts with ultrafast (< 1 ps) pulses. In this experiment, the interaction between two femtosecond pulses and MoS_2_ nanoparticles suspended in liquid is studied. We found that the laser pulses induce bubble formation on the surface of a nanoparticle and a nanoparticle aggregate then forms on the surface of the trapped bubble. The processes of formation of the bubble and the nanoparticle aggregation are intertwined.

## Introduction

The interaction between light and matter in liquids is a field of study that is vital for the advancement of biomedical^1–5^ and optical sensing applications^6–12^. Nanoparticles are especially useful for studying light-matter interactions due to their properties that differ from their macroscopic source. For example, Wang et al., used gold nanoparticles to induce a high-speed liquid flow by inducing an ultrasonic photoacoustic effect using a pulsed laser^11^. With metal nanoparticles, laser pulses have been used to manipulate particles for controlled transport^12^, allowing for localized light absorption for photothermal applications.

Laser ablation and laser-induced agglomeration are two light-matter interaction processes in liquid that are often related. Laser ablation in liquids has been studied extensively for its ability to create nanoparticles, in which a high-energy pulsed or continuous wave laser is focused onto a solid metal target, ablating the target and creating nanoparticles in solution^6–9,13–16^. The cavitation bubble formed in the process is also useful for a variety of applications, especially as a precursor to laser-induced agglomeration of nanoparticles dispersed in liquid. In laser-induced agglomeration, the optical tweezing effect induced by subsequent laser pulses aggregates the suspended nanoparticles to form structures^17^. Depending on the particle properties, such as shape and composition, nanostructures in the liquid medium can be formed. Laser-based agglomeration of nanoparticles has been shown using gold nanoparticles fabricated via laser ablation in liquids^17,18^. The aggregates formed via ablation and agglomeration can be used to enhance nonlinear optical phenomena^19^. However, these techniques are limited in their scale due to the high pulse energy requirement for the synthesis of nanoparticles, with many of these experiments requiring pulse energies in the mJ regime^6–9,13^. The high pulse energy requirement gives these techniques high potential for damage of the surrounding material.

Different approaches have been developed to bypass the high pulse energy requirement for laser-induced agglomeration and minimize damage to surrounding material. One method is to use nanoparticles already suspended in a liquid, which reduces the required laser power, and the size and shape of the nanoparticles can be more accurately controlled prior to aggregation. Another method is to use multiple low-energy laser pulses with different wavelengths as the cavitation bubble formed prior to agglomeration can be optically trapped by the second laser pulse^20,21^. Optical trapping, in which a focused laser beam creates a force gradient that can trap a nanoparticle within the focus of the laser^12^, is a tool that is useful for manipulating nanoparticles and creating structures^18^. However, single laser beam traps have some shortcoming in terms of fine control of the trapped particle. To circumvent this, double-laser optical traps have been developed^22,23^, using two lasers to trap an object. The second laser has been utilized to perform localized excitation for spectroscopic applications such as Raman and fluorescence spectroscopy^23^ as well as to provide finer control of the optical force gradient^22^.

In this paper, we investigate the complex dynamic processes of dual laser-induced agglomeration of suspended nanoparticles in liquid. Molybdenum disulfide (MoS_2_) is a transition metal dichalcogenide that has been extensively studied for its optical^24–27^, electrical^28,29^, and catalytic^30,31^ properties. The presence of a direct excitonic transition^32^ in MoS_2_ allows for the enhancement of nonlinear optical phenomena, making it an attractive candidate as a substrate for optical studies. MoS_2_ nanoparticles in solution have also been used to enhance nonlinear optical phenomena as they were employed to enhance the coherent anti-Stokes Raman scattering intensity of the surrounding pyridine medium, with a calculated nine orders of magnitude enhancement of the Raman excitation^33^. MoS_2_ is also nontoxic in bulk form, shown previously by Weng et al.^34^, making it suitable for optical study of biological samples. However, investigations into how ultrafast (< 1 ps) laser pulses affect the behavior of MoS_2_ nanoparticles in fluids have not been performed, with experiments only performed on mono- and few-layer nanosheets^35^. These investigations are vital to understanding potential interactions that may occur during the integration of multiple femtosecond laser pulses of differing wavelengths with MoS_2_ nanoparticle solutions.

Here, we image and study the interaction between two low-energy femtosecond laser pulses and suspended MoS_2_ nanosheets in an ethanol solution. We observed the intertwined interaction of the laser-induced formation of a cavitation bubble and the subsequent agglomeration of MoS_2_ nanoparticles. Two focused laser pulses—one narrowband pump pulse centered at 800 nm and one broadband supercontinuum pulse (800–1000 nm)—create a cavitation bubble when a nanoparticle passes through the focal point. As the bubble grows, MoS_2_ nanoparticles in the solution agglomerate onto its surface, creating a nanoparticle aggregate. Using suspended nanoparticles instead of a metal or semiconductor target reduces the required power for bubble formation and subsequent aggregation. Other nanoparticles, including Al nanosheets and polystyrene beads, were also studied using this scheme.

## Results

### Laser-induced cavitation and nanoparticle aggregation

**Figure 1:**
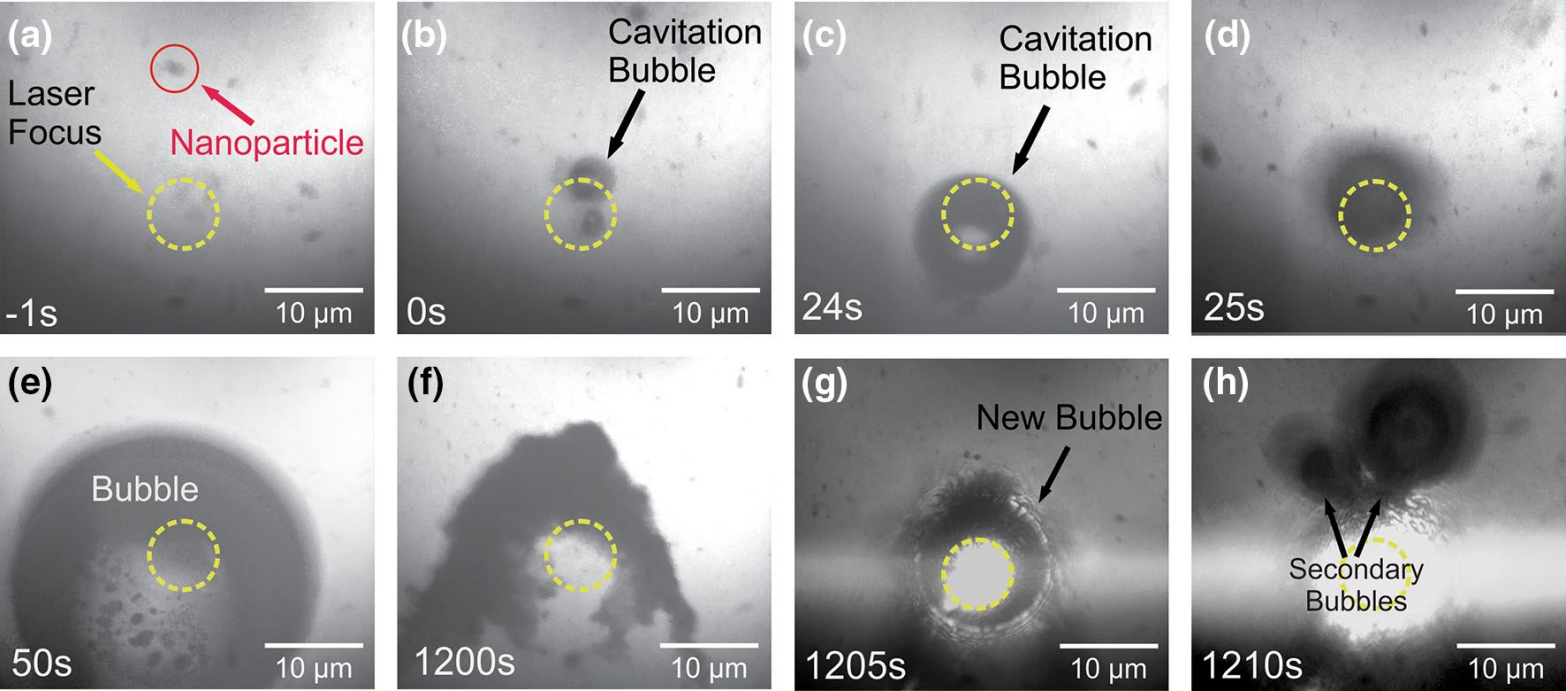
Laser-induced bubble formation and nanoparticle aggregation process. Times are relative to initial bubble formation. (**a**) A nanoparticle (red circle) flows towards the focused spot of the laser (yellow dotted circle). (**b**) A cavitation bubble is formed when the nanoparticle enters the laser focus. The bubble is small (~ 5 µm diameter) and not centered in the laser focus. (**c**) The bubble has increased in size but is still not centered in the focus. (**d**) The bubble becomes centered in the laser focus and also becomes defocused from the camera’s focal region. (**e**) Bubble grown and nanoparticles can be seen on the surface of the bubble. (**f**) The bubble has fallen away from the focus and the nanoparticle aggregate is left behind. (**g**) A new bubble forms in the focus with the aggregate attached. (**h**) Trapped ethanol within the aggregate forms additional bubbles. Laser powers are 16 mW and 8 mW for the supercontinuum and pump pulses, respectively.

Figure 1 shows interaction between the incident laser pulses and MoS_2_ nanoparticle as the particle passes through the laser focus. The incident power for the pump and supercontinuum pulses are 16 mW and 8 mW, respectively. The formation of the bubble begins when a particle (red circle in Fig. 1a) enters the focus (yellow circle) of the incident laser pulses. The nanoparticle enters the focus due to the optical tweezing effect, in which focused laser pulses or beams create a force gradient trapping particles^12^. Once in the laser focus, the bubble is formed on the surface of the nanoparticle but outside of the focus spot (Fig. 1b) due to a process similar to laser ablation in liquids^36^, except the bubble does not burst due to a lack of cavitation pressure and is allowed to grow in liquid. In the first short period (~ 25 s) of the lifetime of the bubble, the bubble sits just outside of the laser focus (Fig. 1c). The collision of the downward flow of the nanoparticles with the bubble in solution is counteracting the optical tweezing effect induced by the incident laser pulses. As a result, the bubble repeatedly moves into and out of the bubble focus. During this period, bubble growth is slow, with the diameter growing from approximately 5 μm to 9.5 μm in 45 s for the bubble depicted in Fig. 4, an increase in diameter of 90%. Nanoparticles begin to attach onto the bubble as they flow towards the focus. After this “out-of-focus” growth period, the bubble then shifts into the focus (Fig. 1d). The bubble is able to enter the focus because its mass is large enough that the nanoparticle collisions do not impart a large enough change in bubble’s velocity to move it out of focus. When the bubble enters the focus bubble growth is more rapid than the out-of-focus period. In a similar time interval to that of the out-of-focus period, the bubble diameter increases from 9.5 to 26.5 µm, an increase of 178% in diameter (Fig. 1e). The rate at which nanoparticles attach onto the bubble also increases compared to the out-of-focus period. Once the bubble becomes too heavy or the incident laser pulses are blocked, the bubble falls off, leaving the aggregate created by the attached nanoparticles in the laser focus (Fig. 1f), and the process repeats. In most situations, the aggregate is then left behind in the focus; otherwise, the aggregate stays attached to the bubble as it falls out of the focus. In the former case, the presence of the aggregate in the laser focus immediately forms a new cavitation bubble in the focus, bypassing the out-of-focus period (Fig. 1g). Secondary bubbles also form due to trapped solvent within the aggregate (Fig. 1h). These secondary bubbles can then merge with the primary in-focus bubble and further accelerate the bubble growth. The growth process then continues, with either further growth of the nanoparticle aggregate or, in the case that the initial aggregate falls away with the bubble, formation of a new aggregate. An additional timeline of bubble formation for a lower combination of laser power (14.0 mW pump and 10.5 mW SC) is available in the Supplementary Fig. S1. Temporal overlap of the two lasers is necessary for bubble formation until the SC power is 27 mW, as no bubble formation was observed until the two lasers are close to maximally overlapped as observed through the BBO crystal.

### Laser-induced bubble rotation and convection

**Figure 2:**
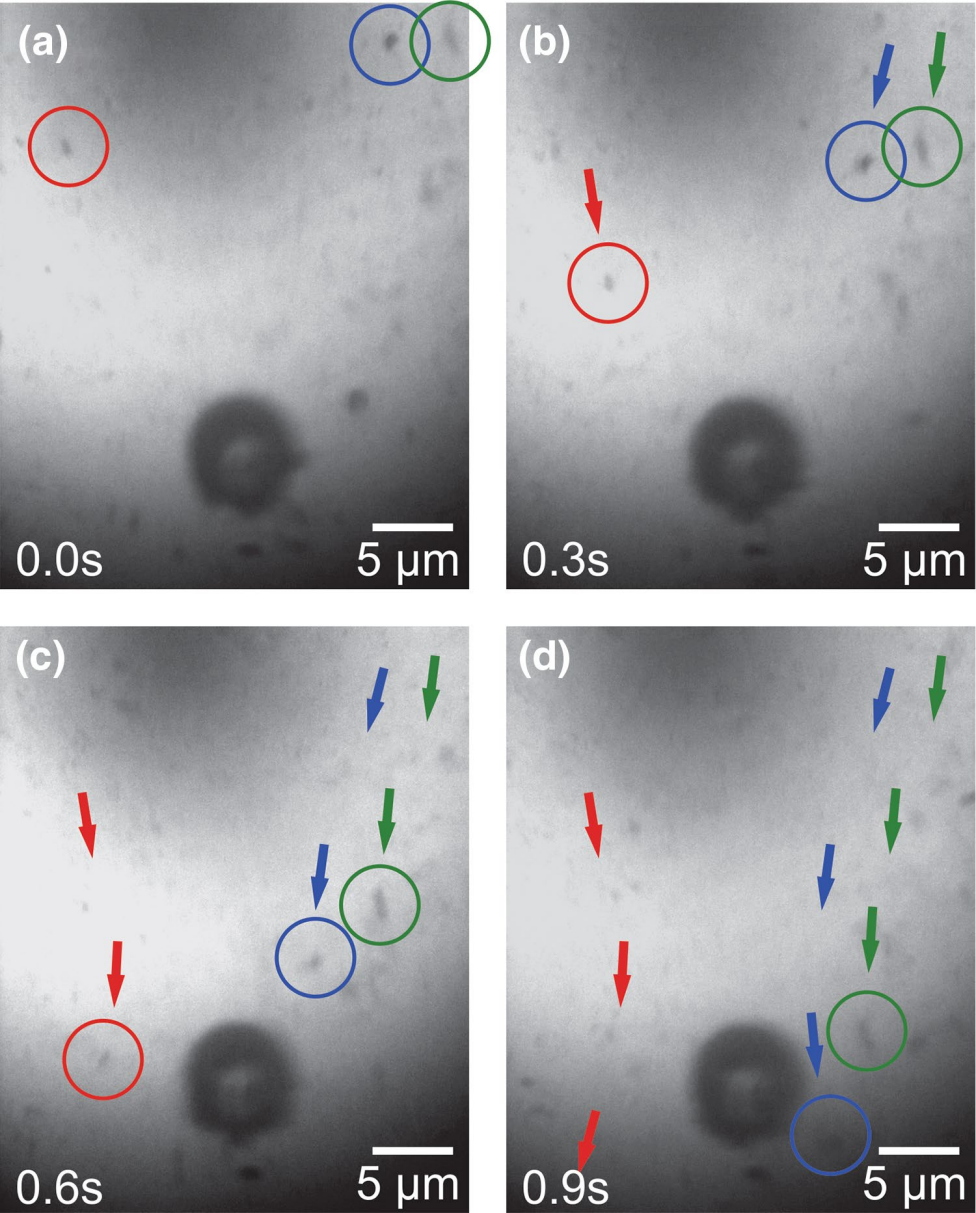
Convection current created by a laser-induced cavitation bubble. Frame-by-frame images of nanoparticle movement in the presence of a laser-induced cavitation bubble. Time between frames is approximately 0.3 s. Arrows indicate motion from the previous frame. An animation of the convection current can be viewed in the Supplementary Video S1.

Video recordings of the entire process show that the formation of the nanoparticle aggregates on the bubble is related to the convection currents that are generated as the bubble is formed and grows in size, both while out of the focal point and in the focal point. The flow of the nanoparticles in solution follows the convection currents. Figure 2 shows the path of the nanoparticles near the bubble by monitoring the positions of the particles using a video camera. An animation depicting frame-by-frame movement of the nanoparticles is available in Supplementary Movie S1. The particles travel along a curved path when the bubble and laser pulses are present. The currents are created due to the heating of the bubble by the incident laser pulses. This creates a difference in surface tension between the bubble and the surrounding solvent, creating convection currents within the solvent^37^. The nanoparticles travel along the convection currents towards the bubble and attach themselves to the bubble. Supplementary Fig. S2 shows a nanoparticle attaching onto the bubble. In the first frame (Supplementary Fig. S2a), the nanoparticle is suspended in the liquid and flows along the convection currents towards the bubble. In the second frame (Supplementary Fig. S2b), the nanoparticle attaches onto the bubble. As the particle attaches to the bubble, it imparts torque onto the bubble, causing the bubble to rotate, as shown in Supplementary Fig. S2c, d. The increased rotation of the bubble also further increases the speed of the convection currents, increasing the frequency of nanoparticle collisions onto the bubble surface.

### Nanoparticle aggregation

**Figure 3:**
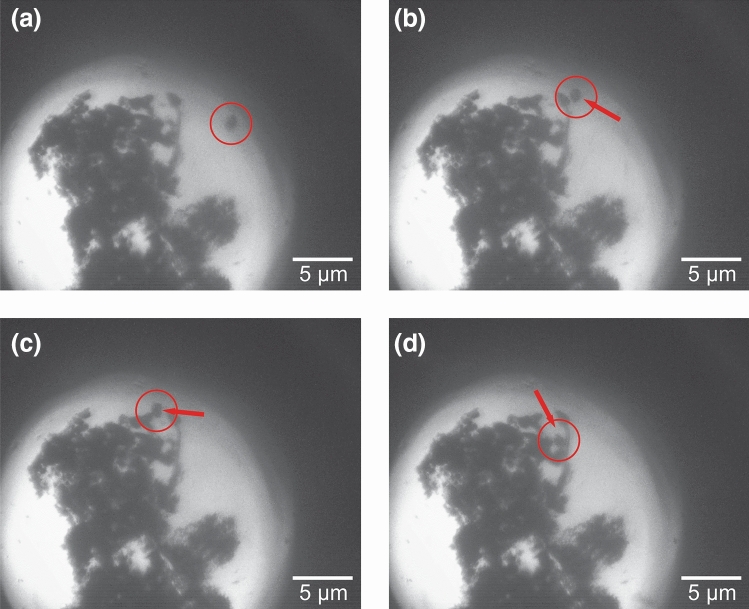
MoS_2_ nanoparticle aggregation on a cavitation bubble. Selected frames of an MoS_2_ nanoparticle attaching onto a nanoparticle aggregate formed on the surface of a cavitation bubble. The nanoparticle is tracked with the red circle and the arrow indicates motion of the nanoparticle from the previous frame. Time between frames is approximately 0.3 s. An animation depicting all frames of this event is available in the Supplementary Video S2.

As particles attach onto the surface, the optical tweezing effect of the incident laser pulses manipulates the nanoparticles to aggregate within the beam path. Figure 3 depicts a nanoparticle aggregate on the surface of the bubble and the motion of a nanoparticle attaching to the aggregate. The images are taken over the course of 11 frames, with the time between frames is approximately 0.15 s. An animation is available in Supplementary Movie S2. The nanoparticle aggregate is centered at the laser focus, with the dark background corresponding to the portion of the bubble where light is not transmitted through to the camera, as seen previously in Fig. 1e. The nanoparticle, highlighted by the red circle in Fig. 3, begins near the dark background. Over the course of the animation, the particle moves, via the optical tweezing effect, towards the center until it attaches to the nanoparticle aggregate. Once attached, the particle moves along with the larger aggregate.

### Pulse dependence

**Figure 4:**
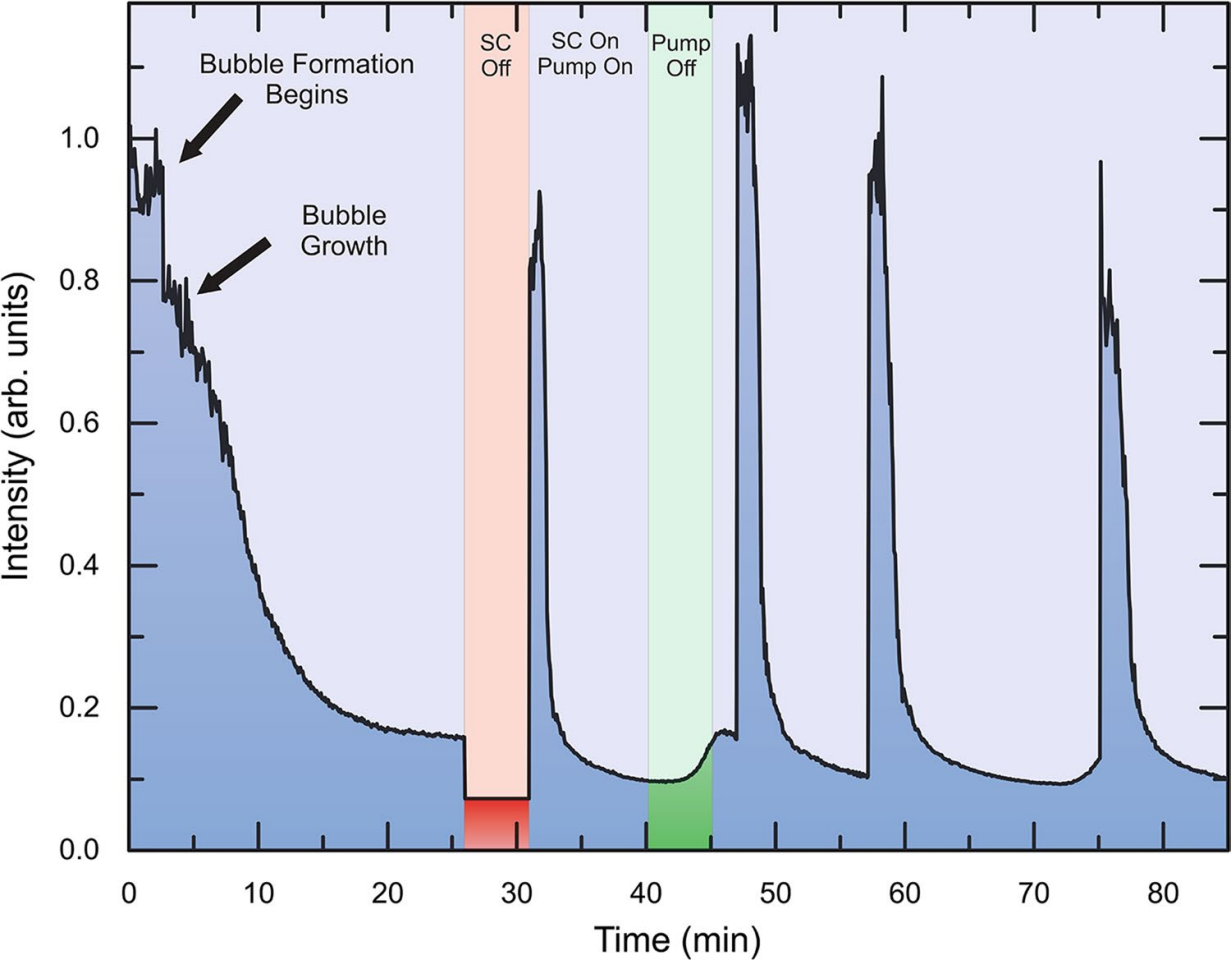
Supercontinuum intensity measurement and pulse dependence. Supercontinuum (SC) intensity measurement performed to determine the effects of individual pulses on the bubble formation process. Bubble formation is characterized by a sharp decrease in the SC intensity. When the SC pulse is blocked (red region), the SC intensity increases indicating that the bubble has fallen away from the focus. Blocking the pump pulse (green region) shows that the bubble still remains in the focus. Laser powers used are 27 mW and 6.1 mW for the SC and pump pulses, respectively.

Supercontinuum intensity measurements were performed to observe the overall bubble formation process on a longer timescale as well as investigate the difference in effects of the two pulses on the process (Fig. 4). Measurements were conducted by removing one of the short pass filters after the sample and measuring the transmitted intensity of the supercontinuum pulse. A decrease in measured signal indicates the formation of a cavitation bubble, as the transmitted light is scattered away from the original path by the bubble. Similarly, an increase in the measured supercontinuum intensity correlates to the absence of a bubble in the beam focus. To observe the effects of individual pulses on the interaction, a beam block is placed in either the 800 nm pump line or the broadband supercontinuum line to prevent it from reaching the sample. When the sample is first placed in the laser path (t < 3 min), the measured supercontinuum signal oscillates due to nanoparticles that are passing through the laser path without forming a bubble. Shortly after, there is a sharp decrease in the supercontinuum intensity, signaling the bubble formation process. As the bubble grows, a larger portion of the supercontinuum light is scattered away from the normal optical path, resulting in a lower transmitted signal. The center of the bubble also begins to scatter light away from the optical path as nanoparticles aggregate on the surface. When the supercontinuum pulse is blocked for five minutes (red region of Fig. 4, t ~ 26–31 min), a sharp increase in the intensity is observed after the supercontinuum is unblocked, meaning that the bubble is no longer in the focus and the light passes through. However, after a few minutes, the supercontinuum intensity decreases, indicating that a new bubble has formed in the focus. When the pump pulse is blocked instead (t ~ 40–45 min, green region of Fig. 4), the intensity of the supercontinuum pulse stays low, indicating that the bubble is still in the focus. The slight increase in SC intensity in the green region can be attributed to a reduction in tweezing force from the absence of the pump pulse causing nanoparticles to fall off the surface of the bubble. Similar experiments show that the effect of the supercontinuum pulse is more significant than that of the pump in trapping the bubble in place, as the bubble remains in the laser path while the pump pulse is blocked. In these supercontinuum measurements, we observe a periodic behavior of bubble formation and bubble fall off as the bubble increases in size. The bubble lifetime period appears to be approximately 15 min. An additional measurement at different pulse powers is depicted in Supplementary Fig. S3.

**Figure 5:**
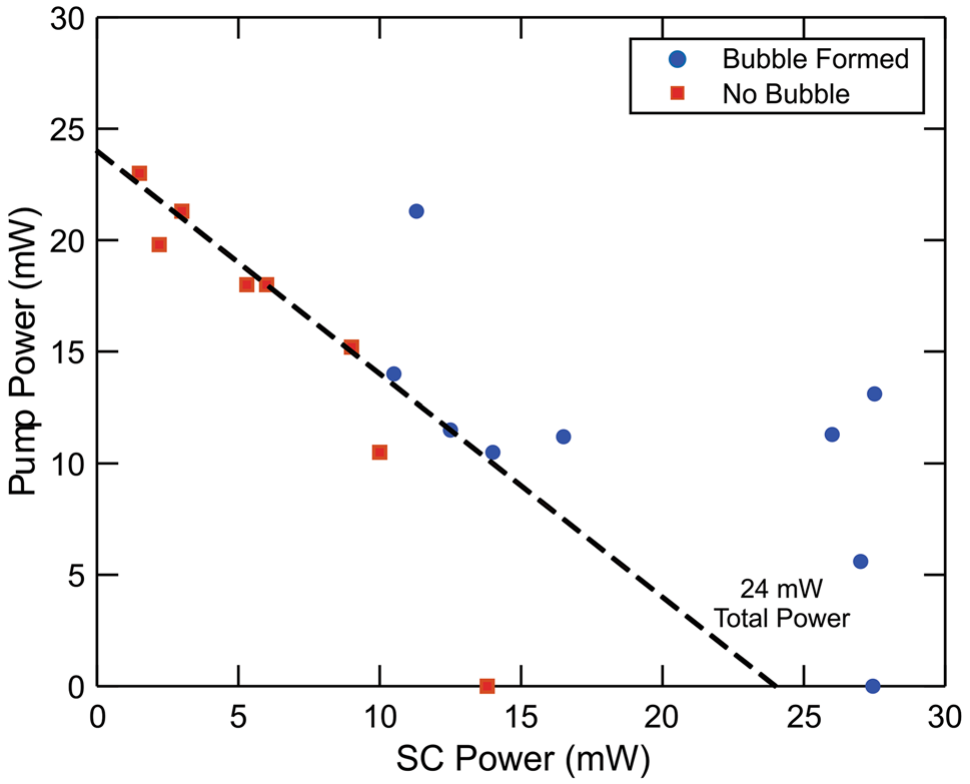
Bubble formation outcomes for different pulse power combinations. Scatter plot of different power combinations between the narrowband pump and broadband supercontinuum (SC) pulses to determine pulse dependences in bubble formation. Power combinations that resulted in bubble formation are denoted as blue circles, while power combinations in which no bubble was observed are depicted as red squares. The black dotted line represents 24 mW of combined laser power. This black dotted line signifies that bubble formation is more reliant on the SC power than the pump power.

The experiment was conducted with different power combinations between the two incident pulses (pump and SC) to investigate the difference in their effects on the behavior of the bubble. Measurements were performed by placing two neutral density filters, one in each pulse path, in order to control the power of the pulses without modifying their spectral character. The laser properties (center wavelength and bandwidth) of the Ti:Sapphire oscillator were modified in order to control the proportion of pulse power that is reflected by the 3 nm bandpass filter into the photonic crystal fiber to create the supercontinuum pulse while fixing the wavelength of the 800 nm narrowband pump pulse. Pulse path lengths were adjusted with each measurement to ensure maximal temporal overlap between the pulses. The dependence of bubble formation on the individual pulse power is plotted in Fig. 5. Pulse power combinations that resulted in the formation of a bubble are denoted as blue circles, while those that did not form a bubble are shown as red squares. The dashed line in Fig. 5 corresponds to 24 mW of combined pump and SC pulse power. If the effects of the pump and SC pulses were equivalent, the outcome of bubble formation would not change moving along lines of constant power. Because the outcome changes along the dashed line, the bubble formation process is more dependent on one pulse than the other. Moreover, because bubble formation is not observed along the dashed line in Fig. 5 at power combinations with a high pump power contribution, the SC pulse has a larger effect on bubble formation than the pump pulse. The presence of successful bubble formation at 27 mW SC and 0 mW pump power also confirms the greater dependence on the SC pulse.

Statistically, 40 bubble formation events over 8 separate solution samples were measured using a SC power of approximately 27 mW and a pump power of about 10 mW. Of those 40 bubble formation events, the lifetime of each event varied from approximately 15–25 min, with the first bubble formation event in a sample set having a longer lifetime than subsequent bubbles.

## Discussion

### Pulse dependence of bubble formation

From the SC intensity measurements and the pulse power dependence measurements, the SC pulse contributes more to the bubble formation and nanoparticle aggregation processes than the narrowband 800 nm pulse. One possible reason for the greater dependence on the SC pulse is that the tweezing force applied by the SC pulse is greater than that of the 800 nm pulse. The tweezing force gradient applied by a laser pulse with pulse duration τ can be approximated as^38^:

1 $$F_{pulse} = - \frac{2E}{{\tau \sqrt \pi }}\frac{z}{{\left( {1 + \left( {z/z_{s} } \right)^{2} } \right)^{2} }}\exp \left( { - \left( {\frac{t}{\tau }} \right)^{2} } \right){\exp}\left( { - \left( {\frac{z}{{z_{s} }}} \right)^{4} } \right),$$

where E is proportional to the pulse energy per cross-section area, and z_s_ = πω_0_^2^/λ_0_ is a distance parameter. From the first term, $2E/\tau \sqrt \pi$, there is an inverse proportional dependence on the pulse duration. From the time-bandwidth product, the 3 nm FWHM bandwidth 800 nm pulse has a minimum pulse duration of 313 fs. The SC pulse generated by the photonic crystal fiber has a much shorter pulse duration, approximately 100 fs, due to the near-zero dispersion of the PCF. As a result, the maximum amplitude of the tweezing force from the SC pulse is about three times greater than that of the narrowband 800 nm pulse. Thus, the 800 nm pulse alone is unable to trap the bubble in the laser focus, preventing the bubble from growing.

Due to the complex nature of dual-laser bubble formation, many factors can affect the formation and lifetime of the bubble^39^, as evidenced by the variation in lifetime in the SC transmission intensity measurements shown in Fig. 4. Factors such as the increase in pressure caused by the formation of the cavitation bubble^40,41^, changes in the optical force gradient^22^, and the surrounding medium can affect the bubble formation^42^. Differences in individual laser powers as well as the spatial and temporal overlap of the two pulses can change the profile of the force gradient of the optical trap and affect the heating of the bubble. The bubble itself can refract the pulses and affect the overlap of the pulses, changing the energy absorption efficiency. Moreover, there is a distinct difference between the first bubble formation event and the ensuing bubble formation events within a sample set, where the formation of the first bubble takes a longer time than the second or the following bubbles. This is attributed to the presence of an aggregate at or near the focal point of the laser, which guarantees that a particle is in the focus to induce the formation of a cavitation bubble.

### Bubble heating

The temperature on the surface of a nanoparticle after laser irradiation of two femtosecond pulses was calculated using the method shown by Burgess et al.^43^. The calculations can be found in the Supplementary Information Discussion S1. The calculations found that the surface temperature on an MoS_2_ nanoparticle is approximately 5370 K, well above the threshold for ethanol evaporation. It also shows the role of the 800 nm pump pulse, in that MoS_2_ has a higher absorption coefficient at 800 nm than in the IR regime of the SC, allowing for more efficient heating of the bubble than with just the SC pulse alone. However, after initial bubble formation, due to the lensing effect of the bubble on the laser light as well as the pulsed nature of the irradiation, the irradiated spot cools down between pulses, preventing optical breakdown of the nanoparticles trapped on the surface of the bubble.

### Comparison with other nanoparticles

The bubble formation experiment was performed using nanoparticles of different geometries and compositions in order to determine the cause of bubble formation and nanoparticle aggregation. The results of these experiments are shown in the Supplementary Information in Supplementary Table S1. From the table, only aluminum nanosheets with size of about 1 micron, were able to create a suspended bubble in the laser focus. Thus, a flat flake shape with a span on the order of 1 square micron could create a bubble. This is possibly due to the flat nanosheets acting as a surface like that of the metal targets used in laser ablation in liquids experiments^1,6–9,13^. The dependence on the nanoparticle geometry could imply that the bubble formation process is possible with other nanoparticle compositions if the nanoparticles are two-dimensional. Absorption spectra for each nanoparticle were also taken to determine if the bubble formation event has a plasmonic origin. The absorption spectra can be found in the Supplementary Information in Supplementary Fig. S4. From the absorption spectra, there is no plasmonic origin to the bubble formation as both MoS_2_ and aluminum do not have an absorption peak at the wavelengths of the laser pulses.

The observed MoS_2_ nanoparticle aggregation can be used in nonlinear optical studies as a potential enhancer of optical signals such as Raman scattering by increasing the amount of analyte-surface contact within the focal point of the laser. The increased analyte-surface contact can also be used to increase light-based catalysis between the nanoparticle and the sample. The low pulse energy requirements (< 1 nJ combined pulse energy) of this aggregation scheme, which does not require the ablation of a metal target which requires pulse energy on the order of mJ^6–9^, make it possible to use with photo-sensitive samples with reduced chance of damaging the sample. Because of the capability of MoS_2_ to enhance nonlinear optical phenomena and its nontoxicity in bulk form, MoS_2_ nanoparticle aggregates can be especially useful in biological applications, such as creating localized points of increased light absorption. In this study, we have demonstrated the above technique can be done using MoS_2_, allowing for the use of light-assisted transport of MoS_2_ nanoparticles for applications such as nanostructure fabrication for samples that are incompatible with traditional metal substrates. The overall bubble formation process is similar to the donut-shaped object that was observed by Zhang et al., using gold nanoparticles on glass in liquid^18^. However, Zhang and co. suggest that the donut-shaped object is a nanoparticle aggregate. In our experiment, due to the merging event shown in Fig. 1G, we determine that the donut-shaped object is a bubble, with the dark ring seen in the image caused by the scattering of incident light away from the camera.

## Conclusion

The interaction between MoS_2_ nanoparticles and ultrafast laser pulses was studied. Laser-induced aggregation of suspended MoS_2_ nanosheets in an ethanol solution was observed using two low-power ultrafast pulses—one narrowband pulse centered at 800 nm and a broadband supercontinuum pulse with wavelength from 800 to 1000 nm. The pulses induce heating of the surrounding ethanol solvent when a nanoparticle passes through the focal point, creating a bubble. The bubble is then heated by the incident laser pulses to create convection currents that draw nanoparticles towards the bubble. Optical tweezing traps the bubble and nanoparticles into the laser focus, where aggregation occurs. When the bubble falls off, the aggregate is left behind to be used. The process is dependent on the geometry of the nanoparticles, with only flat nanoflakes producing a bubble and forming an aggregate. This aggregation scheme can be used as an alternative to coinage metals such as gold and silver in nanoparticle aggregation studies for use with sensitive samples.

## Materials and methods

**Figure 6:**
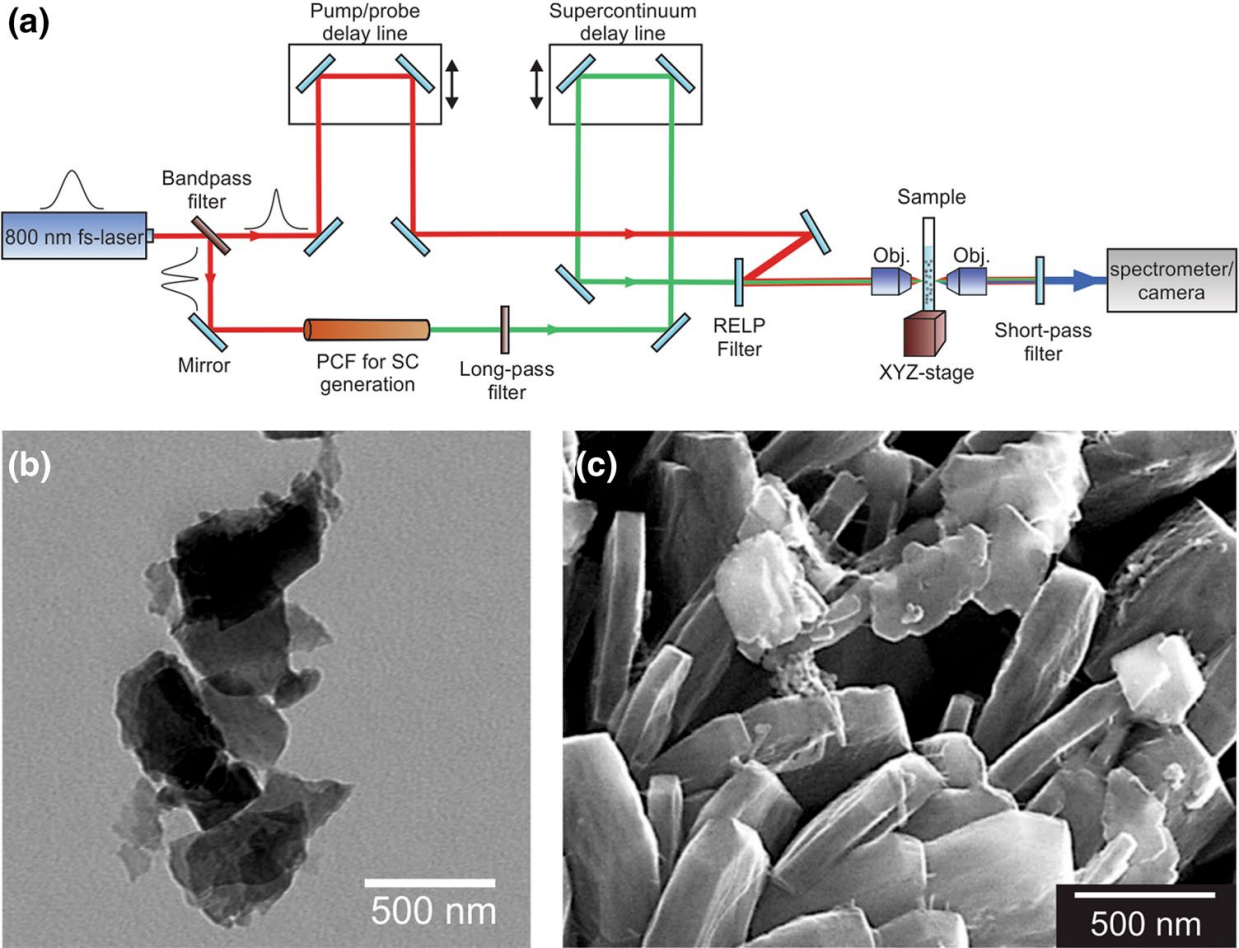
Experimental setup and nanoparticles. (**a**) Experimental setup. A femtosecond laser (800 nm, 100 fs, 30 nm FWHM) is split into two pulses using a bandpass filter. The narrow, transmitted pulse supplied the 800 nm pulse, while the reflected light pumps a photonic crystal fiber to create the broadband supercontinuum pulse. (**b**) Transmission electron microscope of homemade MoS_2_ nanoflakes. (**c**) Scanning electron microscope of homemade Al nanoparticles.

To study the interaction between light and the nanoparticles suspended in liquid, the experiment was conducted using a broadband femtosecond coherent anti-Stokes Raman spectroscopy setup^44^ (Newport CARS-KT) depicted in Fig. 6a. A 532 nm Nd:YAG laser (Spectra Physics Millenia eV) pumps a Ti:Sapphire oscillator (Spectra Physics Tsunami). The oscillator has a repetition rate of 80 MHz and is mode-locked to be a Gaussian pulse centered at 800 nm with a bandwidth of approximately 30 nm FWHM and the pulse duration to be ~ 100 fs. The laser pulse is then split using a 3 nm bandpass filter. The transmitted, narrow-band pulse continues towards the sample. The remaining bandwidth of the oscillator pulse is reflected off the bandpass filter and focused onto a photonic crystal fiber (Newport PCF-800). The photonic crystal fiber creates a supercontinuum pulse from 500 to 1100 nm. A long-pass filter then cuts off wavelengths below 785 nm to form the near-infrared supercontinuum pulse. The narrowband pump pulse and the broadband supercontinuum are then recombined using an 800 nm razor-edge long pass filter (RELP) and directed towards the sample. Neutral density filters are used to modulate pulse energy, with energies typically less than 1 nJ for each pulse. Spatial and temporal overlap are achieved by redirecting the pulses onto a BBO crystal and modifying individual path lengths until a four-wave mixing signal is observed, signifying that the pulses are aligned spatially and temporally. Once spatial and temporal overlap are achieved, the pulses are redirected back towards the sample. The signal is then filtered using two short pass filters to remove the incident pulses. Video and images were taken using a CCD camera (IDS uEye 2240) placed after the filters. For taking the image with the camera, a white light source was placed before the sample and directed using a glass slide to the sample collinear to the laser pulses. Supercontinuum spectra for intensity measurements were collected using a spectrometer (Horiba iHR 550).

MoS_2_ nanosheets were prepared using the lithium-intercalation/exfoliation method^45^. First, 0.5 g MoS_2_ powder was immersed in 5 ml of 1.6 M *n*-butyl lithium solution (0.1 g/ml) and stirred for 48 h in an argon protected container to produce Li-intercalated MoS_2_ (Li_x_MoS_2_). Li_x_MoS_2_ was filtered and washed repeatedly with hexane to remove excess lithium and organic residues. Then the Li_x_MoS_2_ powder was dispersed in 100 ml Nanopure™ water (18.3 MΩ). Subsequently, Li_x_MoS_2_ was exfoliated via bath ultrasonication and the MoS_2_ nanosheets were separated through centrifugation. The nanosheets are then transferred into a clean cuvette and placed in a solution of pure ethanol. Scanning electron microscope (SEM) images of the prepared MoS_2_ nanosheets are shown in Fig. 6b. The nanosheets are irregular flakes in shape with an average size of 300–400 nm and thickness of several layers (~ 10 nm). Aluminum nanosheets^46^ were prepared via ultrasonication of commercial aluminum foil in ethylene glycol. The Al nanosheets were separated through centrifugation to remove the ethylene glycol and placed in a solution of pure ethanol. An SEM image of the Al nanoparticles are shown in Fig. 6c. The Al nanosheets are irregular sheets. The diameters of the Al sheets are ~ 200–1000 nm and the thicknesses are ~ 50–200 nm. The commercial MoS_2_ nanosheets were purchased from 2D Semiconductors in an ethanol solution. The commercial MoS_2_ nanosheets consist of monolayer to few layer of MoS_2_ and have sizes roughly 50 nm in diameter. The two polystyrene bead solutions were purchased from Polysciences, Inc., and are spherical in shape with radii of 1.053 µm and 0.499 µm. The polystyrene bead solutions were diluted with additional ethanol to increase transmission.

Absorption measurements were conducted using a spectrophotometer (Agilent 8453) equipped with deuterium and tungsten lamps. First, a quartz cuvette filled with pure ethanol is used as a blank, then 0.1 ml of nanoparticle solution in ethanol was measured. Because of differences in sample concentration, relative absorption measurements are compared between samples.

## Supplementary information

Supplementary Information.

Supplementary Video 1.

Supplementary Video 2.

## Data Availability

The datasets generated and analyzed during the current study are available from the corresponding author on reasonable request.
