# Supplementary material for: Multi-pulse laser-induced bubble formation and nanoparticle aggregation using MoS2 nanoparticles
Source: Sci Rep. 2020 Sep 25;10:15753. doi: 10.1038/s41598-020-72689-x (PMC7519087; doi:10.1038/s41598-020-72689-x)
Supplement: Supplementary file 1 — Supplementary Information. [file 41598_2020_72689_MOESM1_ESM.docx]

**Supplementary Materials for**

**Multi-pulse Laser-Induced Bubble Formation and Nanoparticle Aggregation Using MoS_2_ Nanoparticles**

Brian A. Ko, Weigang Lu, Alexei O. Sokolov, Ho Wai Howard Lee, Marlan O. Scully, Zhenrong Zhang*

*Corresponding author. Email: [Zhenrong_zhang@baylor.edu](mailto:Zhenrong_zhang@baylor.edu)

This file includes:

Fig. S1. Additional Bubble Formation Timeline for a different power combination

Fig. S2. MoS_2_ nanoparticle attaching onto a cavitation bubble

Fig. S3. Supercontinuum intensity measurement for another power combination.

Supplementary Discussion S1. Calculation of Laser Heating of Bubble

Supplementary Discussion S2. Absorption Spectrum of MoS_2_ Nanoparticles and Other Nanoparticles

Table S1. Bubble formation results for other nanoparticles.

Fig. S4. Absorption spectra for all nanoparticles used in the experiment.

Captions for movies S1 to S2.

References [*1-5*].

**Other Supplementary Material for this manuscript includes the following:**

Movie S1 (.mp4). Animation of convection current generated via laser heating of a bubble.

Movie S2 (.mp4). Animation of MoS_2_ nanoparticle attaching to the nanoparticle aggregate.


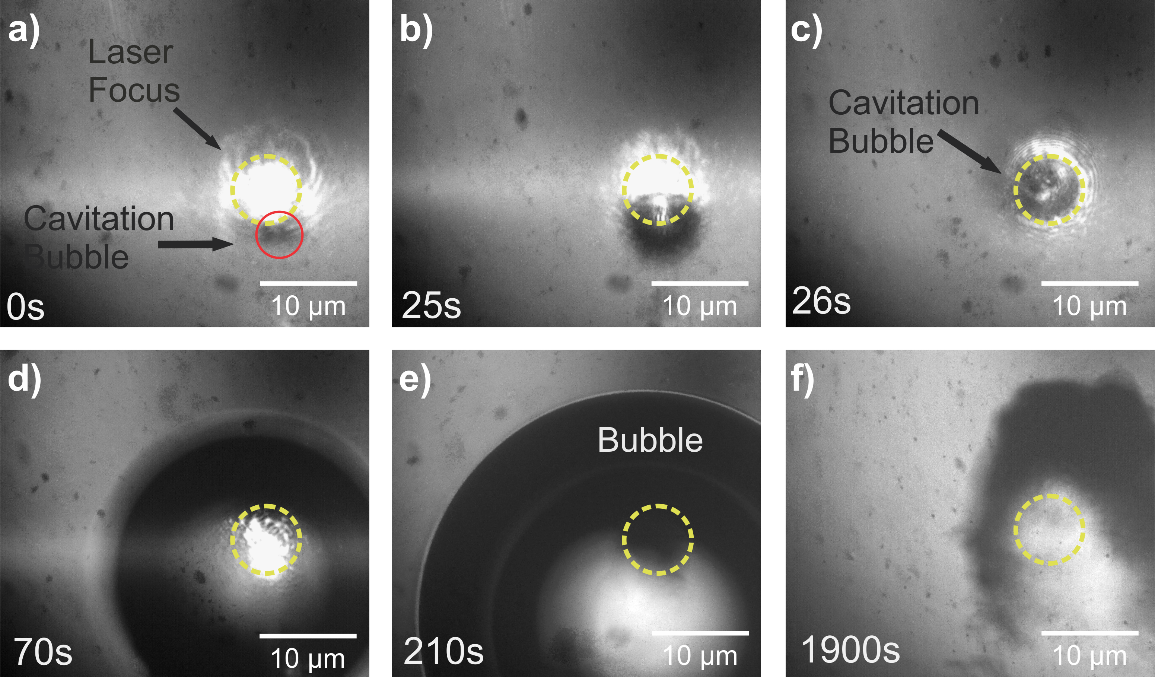
 **Figure S1. Additional Bubble Formation Image**

**Figure S1. Additional timeline of cavitation bubble formation and nanoparticle aggregation of MoS_2_ nanoparticles.** Optical camera images captured at different times showing the light-matter interaction between MoS_2_ nanosheets and two femtosecond laser pulses. Times are relative to initial bubble formation. (a) A nanoparticle (red circle) flows towards the focused spot of the laser (yellow dotted circle). (b) A cavitation bubble is formed when the nanoparticle enters the focus. The bubble is small (~5 µm diameter) and not centered in the focus. (c) The bubble becomes centered in the focus and also becomes defocused from the camera’s focal region. (d) Bubble increases in radius once centered in the focus. (e) Bubble has grown and nanoparticles can be seen on the surface of the bubble. (f) The bubble has fallen away from the focus and the nanoparticle aggregate is left behind. Laser power is 14 mW and 11.2 mW for the supercontinuum and pump pulses, respectively.


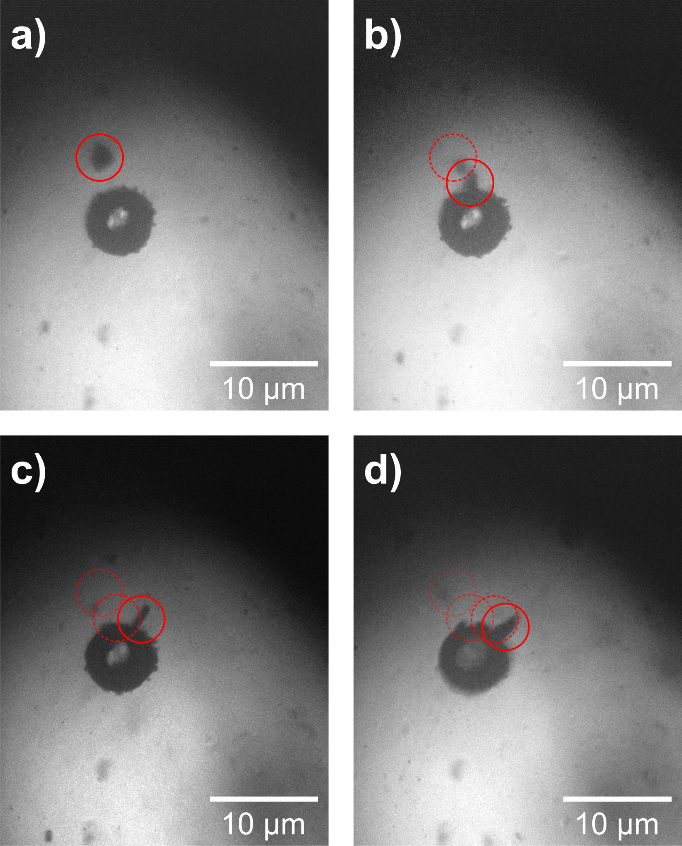
**Figure S2**. **Nanoparticle attaching to bubble and subsequent bubble rotation**

**Figure S2**. **MoS_2_ nanoparticle attaching to a laser-induced cavitation bubble.** An Mos_2_ nanosheet attaches onto the surface of the cavitation bubble. (a) Nanoparticle flows along convection currents towards the bubble. (b) The nanoparticle attaches to the bubble. (c-d) The collision of the nanoparticle imparts angular momentum on the bubble, inducing rotation. Previous locations of the nanoparticle are shown as dotted circles to track the motion.

**Figure S3. Supercontinuum Intensity Measurements for a Different Power
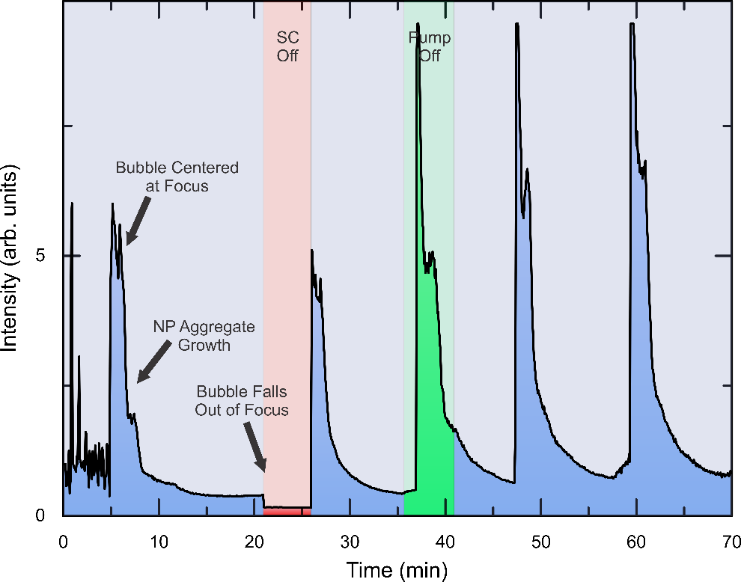
Combination**

**Figure S3**. **Transmitted supercontinuum intensity through a laser-induced cavitation bubble.** Supercontinuum (SC) intensity measurement to detect bubble formation and observe the effects of individual pulses on bubble formation. Laser powers are 26.5 mW and 11.6 mW for the SC and pump pulses, respectively. A decrease in the SC intensity corresponds to the formation and presence of a bubble in the laser path. When the SC pulse is blocked (red region), the bubble falls off, indicated by the increase in SC intensity immediately after the red region. When the pump pulse is blocked (green region), the SC signal is unchanged, though there is a small perturbation in the SC signal intensity.

**Supplementary Discussion S1. Calculation of Laser Heating of Bubble**

The change in surface temperature immediately after irradiation by a square-shaped laser pulse with pulse duration τ is defined as[1]:

$\Delta T\left( \tau\right)=2\left( \frac{F_{0}}{K} \right)\left( \frac{\kappa\tau}{\pi} \right)^{\frac{1}{2}}$ (2)

where F_0_ is the maximum absorbed power density, K is the thermal conductivity of MoS_2_ (2.3 W/m K)[2], and κ is the thermal diffusivity of MoS_2_, defined as:

$\kappa= \frac{K}{\rho C_{p}}$ (3)

where ρ is the density (5.06 x 10^3^ kg/m^3^) and C_P_ is the isobaric specific heat[3] (397.125 J/kg K) of MoS_2_. Substituting these values into equation 3 gives the thermal diffusivity of MoS_2_ as 1.1446 x 10^-6^ m^2^/s.

The maximum absorbed power density by a nanoparticle with dimensions of 1 µm can be calculated from the average power of the incident laser pulses. For the combination of 16 mW of SC power and 8 mW of narrowband pump, the combined absorbed power density can be calculated as:

$F_{0}=A_{SC}I_{SC}+ A_{P}I_{P}$ (4)

where A_k_ is the absorbance of MoS_2_ at the wavelength of the incident light, defined as

$A_{i}=\frac{a_{i}d}{2.303}$ (5)

where d is the thickness of the MoS_2_ nanosheet (~100 nm) and a_i_ is the absorption coefficient of MoS_2_ at the wavelength λ_i_ . For 800 nm, a_p_ = 87422 cm^-1^ and for the wavelength range of the SC pulse, a_SC_ ≈ 60000 cm^-1^ [4]. Thus, A_P_ = 0.3796 and A_SC_ ≈ 0.2605. The total power density on an MoS_2_ nanosheet I_i_, is defined by

$I_{i}= \frac{\bar{P}_{i}}{f\tau A}$ (6)

where $\bar{P_{i}}$ is the average power of the laser pulse, $f$ is the repetition rate of the laser (80 MHz), and A is the beam waist at the focal point (5 µm). Inserting these values into equations 5 and 6 and substituting into equation 4, the maximum absorbed power density F_0_ is

$F_{0}=4.58 \times{10}^{9} W/{cm}^{2}$ (7)

Substituting all values into Equation 2 yields the surface temperature shift of an MoS_2_ nanosheet by a single laser pulse as

$\Delta T\left( \tau\right)=2 \frac{\left( 4.58 \times{10}^{13}\frac{W}{m^{2}} \right)}{\left( 2.3\frac{W}{m K} \right)}\left( \frac{\left( 1.1446 \times{10}^{-6}\frac{m^{2}}{s} \right)\left( 5 \times{10}^{-14}s \right)}{\pi} \right)^{\frac{1}{2}}=5.37 \times{10}^{3} K$ (8)

**Supplementary Discussion S2. Absorption Spectrum of MoS_2_ Nanoparticles and Other Nanoparticles**

Other nanoparticles with varying composition and geometries, in ethanol solution, were tested under similar conditions to understand the mechanism of the formation of the bubble and aggregates due to light-nanoparticle interactions in the liquid. Commercial MoS_2_ flakes (2D Semiconductors) 1 to 10 layers in thickness with sizes on the order of 10 nm were studied to determine if the bubble formation was caused due to the composition of the nanoparticles. Al nanosheets were tested due to its similar geometry and absorption properties to the homemade MoS_2_ nanoparticles – 200-1000 nm span with ~100 nm thickness with a flat rectangular shape and low absorption in the IR regime. Polystyrene spheres (500 nm and 1um diameter) and Ag nanorods (diameter 50 nm, length 1-2 um) with size on the same order to the homemade MoS_2_ nanoparticles were also selected to determine shape dependence. The results of these tests are shown in Table S1. Of the other nanoparticles tested, only the Al nanoparticles with size ranging from 500 nm to 1 um were able to form a bubble under the same experimental conditions. This leads to the conclusion that the geometry of the nanoparticles in the solution, on the order of 1 um and flat shapes, is the deciding factor in bubble formation. The results suggest that the reason that flat particles are necessary for bubble formation is most likely that the bubble forms due to a process similar to laser ablation in liquids. The flat surface of the MoS_2_ and Al nanoparticles functions similarly to that of the metal target material that is present in laser ablation in liquids measurements. The difference is that there is no hard boundary in the nanoparticle situation, so there is no high cavitation pressure that causes the bubble to burst. Instead, the bubble remains in the focal point of the laser pulses and grows as more of the solvent is evaporated.

| Nanoparticle | Shape | Size | | Bubble Formation |
| --- | --- | --- | --- | --- |
| MoS_2_ (homemade) | Nanosheet | 400-500 nm, >10 nm thickness | Yes | |
| MoS_2_ (commercial) | Nanosheet | 10-100 nm, <5 nm thickness | No | |
| Aluminum | Nanosheet | 1-2 µm, 50-100 nm thickness | Yes | |
| Silver | Nanorod | 1-2 µm length, 50 nm radius | No | |
| Polystyrene | Nanosphere | 499 nm radius | No | |
| Polystyrene | Nanosphere | 1.053 µm radius | No | |

**Table S1. Nanoparticles Investigated for Bubble Formation Events**


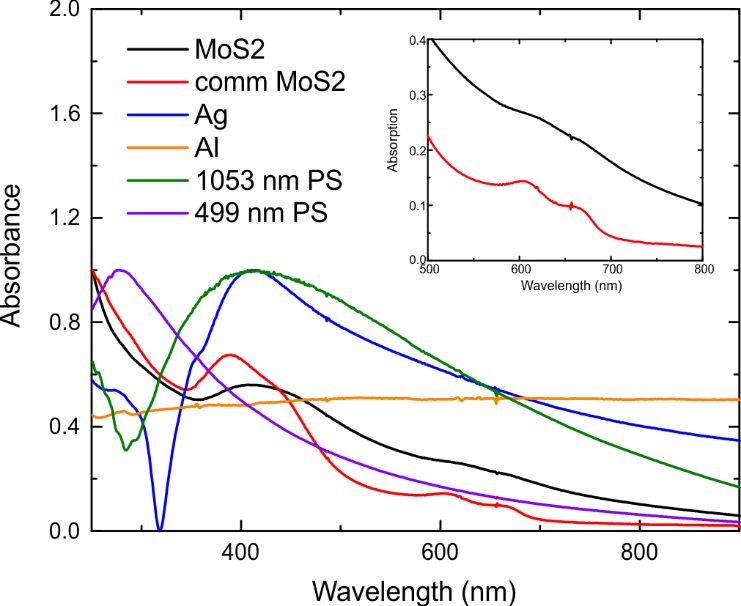
Absorption measurements were conducted in order to determine if the bubble formation is caused related to plasmonic resonances in the nanoparticles. Peaks in absorption spectra indicate a resonance within the material. Absorption measurements for the nanoparticle solutions are shown in Figure S4. For MoS_2_, both homemade and commercial MoS_2_ solutions exhibit absorption peaks at approximately 680 and 600 nm, corresponding to the A and B excitons, respectively[5]. They also have a broader absorption peak at approximately 400 nm, corresponding to the C and D excitons. Absorption measurements of the aluminum nanoparticle solution show that the nanoparticles have a relatively flat absorption spectrum with little features in the visible to near-infrared regime. Ag nanorods show a plasmonic absorption peak at approximately 400 nm with a long tail in the visible regime. 500 nm diameter polystyrene beads exhibit an absorption peak at approximately 300 nm with a long tail in the visible. The 1 um polystyrene beads exhibit a broad absorption peak centered at 418 nm. Because the commercial MoS_2_ nanoparticles did not create a cavitation bubble while having an identical absorption spectrum to the homemade nanoparticles, it can be determined that the bubble formation did not occur due to a resonance. If the bubble formation was caused by a plasmonic resonance, then both the homemade and the commercial MoS_2_ solutions would have produced a cavitation bubble. This is further confirmed with the lack of an absorption peak in the visible or near-infrared regime for the aluminum nanoparticles. This leads to the conclusion that the bubble is formed via the rapid heating of nanoparticles and that the geometry (sheet-like) of the nanoparticle is essential to bubble formation. Because the geometry of the nanoparticle is the most essential factor to this process, it is possible other nanoparticles can be used in a similar manner if they have the same geometry.

**Figure S4.** **Absorption spectra of nanoparticles used in the experiment.** MoS_2_ absorption peaks corresponds to the A (680 nm) and B (600 nm) excitons are shown in the inset for the commercial (red line) and homemade (black line) solutions.

**Supplementary Movie Captions**

**Movie S1. Animation of Convection Current Generated via Laser Heating of a Bubble.** Video animation showing the travel of MoS_2_ nanoparticles suspended in an ethanol solution near a laser-induced cavitation bubble. The heating of the cavitation bubble creates differences in surface tension near the surface of the bubble, inducing convection currents that the nanoparticles flow along. Colored circles highlight the nanoparticles and the arrows mark their displacement from the previous frame. Animation is taken over the course of 1.2s at 8 frames/second.

**Movie S2**. **Animation of MoS_2_ Nanoparticle Attaching to the Nanoparticle Aggregate.** Video animation of Figure 3, in which an MoS_2_ nanoparticle attaches onto the nanoparticle aggregate. The red circle indicates the position of the nanoparticle and the red arrows denote the displacement from the previous frame. Video was taken at 8 frames/second.

**References**

1 Burgess, D., Stair, P. C. & Weitz, E. Calculations of the surface temperature rise and desorption temperature in laser‐induced thermal desorption. *Journal of Vacuum Science & Technology A: Vacuum, Surfaces, and Films* **4**, 1362-1366 (1986).

2 Gandi, A. N. & Schwingenschlögl, U. Thermal conductivity of bulk and monolayer MoS2. *EPL (Europhysics Letters)* **113**, 36002 (2016).

3 Lindstrom, P. J. & Mallard, W. G. (National Institute of Standards and Technology, Gaithersburg MD).

4 Polyanskiy, "M. N. Refractive Index Database," https://refractiveindex.info. Accessed on 2019-05-06.

5 Eda, G. *et al.* Photoluminescence from chemically exfoliated MoS2. *Nano Lett* **11**, 5111-5116 (2011).
